# (2*R*)-2-(1,3-Dioxoisoindolin-2-yl)-4-(methyl­sulfan­yl)butanoic acid

**DOI:** 10.1107/S1600536809028992

**Published:** 2009-07-25

**Authors:** Abdul Rauf Raza, M. Nawaz Tahir, Aisha Saddiqa, Muhammad Danish, M. Saeed Iqbal

**Affiliations:** a Department of Chemistry, University of Sargodha, Sargodha, Pakistan; b Department of Physics, University of Sargodha, Sargodha, Pakistan; c Department of Chemistry, Government College University, Lahore, Pakistan

## Abstract

The title compound, C_13_H_13_NO_4_S, the 1,3-dioxoisoindolin-2-yl unit is planar (r.m.s. deviation 0.0192 Å) and is oriented at a dihedral angle of 79.14 (18)° to the carboxyl­ate group. An intra­molecular C—H⋯O hydrogen bond leads to the formation of a planar (r.m.s. deviation 0.0419 Å)*R*(5) ring motif. In the crystal, mol­ecules are connected through O—H⋯O and C—H⋯O hydrogen bonds with *R*_2_^2^(9) ring motifs into chains extending along the *b* axis.

## Related literature

For the biological activity of isocoumarin and 3,4-dihydro­isocoumarin, see: Hill (1986 ▶); Canedo *et al.* (1997 ▶); Whyte *et al.* (1996 ▶). For related structures, see: Barooah *et al.* (2007 ▶); Feeder & Jones (1994 ▶); Rajagopal *et al.* (2003 ▶). For graph-set motifs, see: Bernstein *et al.* (1995 ▶).
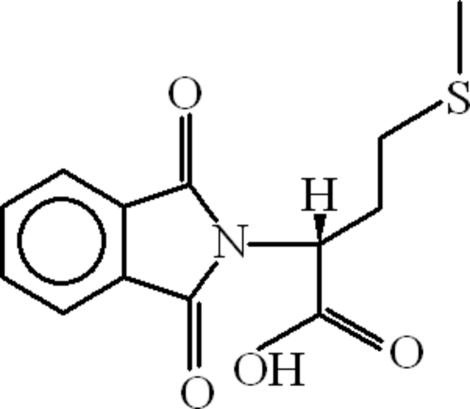


## Experimental

### 

#### Crystal data

C_13_H_13_NO_4_S
*M* *_r_* = 279.30
Orthorhombic, 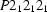

*a* = 6.7923 (6) Å
*b* = 9.9581 (8) Å
*c* = 20.0970 (17) Å
*V* = 1359.3 (2) Å^3^
*Z* = 4
Mo *K*α radiation
μ = 0.25 mm^−1^
*T* = 296 K
0.20 × 0.14 × 0.10 mm

#### Data collection

Bruker Kappa APEXII CCD diffractometer
Absorption correction: multi-scan (*SADABS*; Bruker, 2005 ▶) *T*_min_ = 0.969, *T*_max_ = 0.985
7865 measured reflections
1864 independent reflections
1679 reflections with *I* > 2σ(*I*)
*R*_int_ = 0.024

#### Refinement

*R*[*F* ^2^ > 2σ(*F* ^2^)] = 0.036
*wR*(*F* ^2^) = 0.098
*S* = 1.06
1864 reflections
179 parameters
H atoms treated by a mixture of independent and constrained refinement
Δρ_max_ = 0.20 e Å^−3^
Δρ_min_ = −0.33 e Å^−3^

### 

Data collection: *APEX2* (Bruker, 2007 ▶); cell refinement: *SAINT* (Bruker, 2007 ▶); data reduction: *SAINT*; program(s) used to solve structure: *SHELXS97* (Sheldrick, 2008 ▶); program(s) used to refine structure: *SHELXL97* (Sheldrick, 2008 ▶); molecular graphics: *ORTEP-3 for Windows* (Farrugia, 1997 ▶) and *PLATON* (Spek, 2009 ▶); software used to prepare material for publication: *WinGX* (Farrugia, 1999 ▶) and *PLATON*.

## Supplementary Material

Crystal structure: contains datablocks global, I. DOI: 10.1107/S1600536809028992/pb2001sup1.cif

Structure factors: contains datablocks I. DOI: 10.1107/S1600536809028992/pb2001Isup2.hkl

Additional supplementary materials:  crystallographic information; 3D view; checkCIF report

## Figures and Tables

**Table 1:** Hydrogen-bond geometry (Å, °)

*D*—H⋯*A*	*D*—H	H⋯*A*	*D*⋯*A*	*D*—H⋯*A*
O1—H1⋯O3^i^	0.77 (3)	1.96 (3)	2.673 (3)	154 (3)
C3—H3⋯O2^ii^	0.9300	2.4200	3.328 (4)	165.00
C9—H9⋯O4	0.96 (3)	2.48 (3)	2.905 (3)	106.4 (18)
C11—H11*B*⋯O1^iii^	0.9700	2.5400	3.443 (3)	156.00

Symmetry codes: (i) 
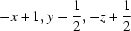
; (ii) 
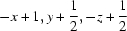
; (iii) 
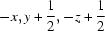
.

**Fig. 1.**
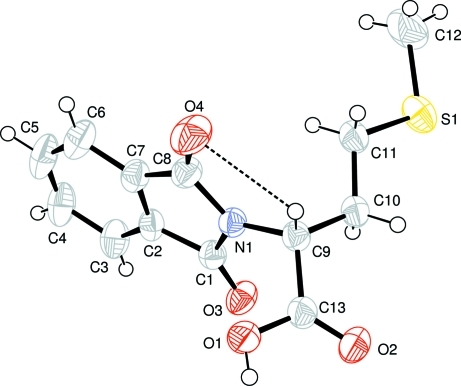
View of the title compound with the atom numbering scheme. The thermal ellipsoids are drawn at the 50% probability level. H-atoms are shown by small circles of arbitrary radii. The dotted line indicate the intramolecular H-bond.

**Fig. 2.**
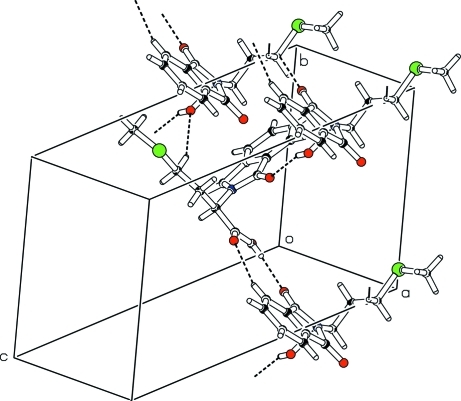
The partial packing (PLATON; Spek, 2009) which shows that molecules form polymeric chains extending along the b-axis.

## supplementary crystallographic information

### Comment

Isocoumarin and 3,4-dihydroisocoumarin have shown an impressive array of biological activities such as anti-tumor (Hill *et al.*, 1986), anti-leucemic (Canedo *et al.*, 1997) and anti-microbial (Whyte *et al.*, 1996). The titled compound (I, Fig. 1) is an intermediate towards the synthesis of chiral isocoumarin. The biological activity of the title compound and synthesis of its complexes are in progress.

The crystal structures of 2-Phthalimidoethanoic acid monohydrate (Feeder & Jones, 1994), N-Phthaloylglycine (Barooah *et al.*, 2007) and DL-Methioninium trichloroacetate (Rajagopal *et al.*, 2003) have been published which contain the moieties of the title compound.

In the title compound the aromatic ring and heterocyclic ring along with O-atoms of carbonyl groups A (C1—C8/N1/O3/O4), the linear chain B (C9—C11/S1/C12) and the carboxylate group C (O1/C13/O2) are planar. There exists an intramolecular H-bond of type C—H···O completing a planar S(5) ring motif (Bernstein *et al.*, 1995). The value of dihedral angle between A/B, A/C and B/C is 80.04 (7)°, 79.14 (18)° and 20.54 (30)°, respectively. Due to the intermolecular H-bonding (Table 1), the molecules are connected in one dimensional polymeric chains through ring motifs R_2_^2^(9) extending along the b-axis.

### Experimental

The methionine (2.0 g, 13.4 mmol) and phthalic anhydride (2.13 g, 14.38 mmol) were added to a flask with constant stirring at 423 K for 2 h. The reaction mixture was brought to room temperature and the crystalline phthallic anhydride on the walls of the flask were removed. The solid crude product was purified by crystallization from ethanol/water (7:3) that afforded colorless prisms of the title compound (I).

Yield 81%.

### Refinement

All the Friedal pairs were merged.

H atoms (for hydroxy and methine) were located in a difference Fourier map and their coordinates were refined. The remaining H atoms were positioned geometrically with C-H = 0.93, 0.97 and 0.96 Å for aromatic, methylene and methyl H atoms, respectively and constrained to ride on their parent atoms, with Uiso(H) = xUeq(C,O), where x = 1.5 for methyl H and x = 1.2 for all other H atoms.

### Crystal data

**Table:**

C_13_H_13_NO_4_S	*F*(000) = 584
*M**_r_* = 279.30	*D*_x_ = 1.365 Mg m^−^^3^
Orthorhombic, *P*2_1_2_1_2_1_	Mo *K*α radiation, λ = 0.71073 Å
Hall symbol: P 2ac 2ab	Cell parameters from 1864 reflections
*a* = 6.7923 (6) Å	θ = 2.3–28.0°
*b* = 9.9581 (8) Å	µ = 0.25 mm^−^^1^
*c* = 20.0970 (17) Å	*T* = 296 K
*V* = 1359.3 (2) Å^3^	Prismatic, colorless
*Z* = 4	0.20 × 0.14 × 0.10 mm

### Data collection

**Table:**

Bruker Kappa APEXII CCD diffractometer	1864 independent reflections
Radiation source: fine-focus sealed tube	1679 reflections with *I* > 2σ(*I*)
graphite	*R*_int_ = 0.024
Detector resolution: 7.50 pixels mm^-1^	θ_max_ = 28.0°, θ_min_ = 2.3°
ω scans	*h* = −8→8
Absorption correction: multi-scan (*SADABS*; Bruker, 2005)	*k* = −12→7
*T*_min_ = 0.969, *T*_max_ = 0.985	*l* = −23→26
7865 measured reflections	

### Refinement

**Table:**

Refinement on *F*^2^	Primary atom site location: structure-invariant direct methods
Least-squares matrix: full	Secondary atom site location: difference Fourier map
*R*[*F*^2^ > 2σ(*F*^2^)] = 0.036	Hydrogen site location: inferred from neighbouring sites
*wR*(*F*^2^) = 0.098	H atoms treated by a mixture of independent and constrained refinement
*S* = 1.06	*w* = 1/[σ^2^(*F*_o_^2^) + (0.057*P*)^2^ + 0.2154*P*] where *P* = (*F*_o_^2^ + 2*F*_c_^2^)/3
1864 reflections	(Δ/σ)_max_ < 0.001
179 parameters	Δρ_max_ = 0.20 e Å^−^^3^
0 restraints	Δρ_min_ = −0.33 e Å^−^^3^

### Special details

**Table:**

Geometry. Bond distances, angles etc. have been calculated using the rounded fractional coordinates. All su's are estimated from the variances of the (full) variance-covariance matrix. The cell esds are taken into account in the estimation of distances, angles and torsion angles
Refinement. Refinement of *F*^2^ against ALL reflections. The weighted *R*-factor *wR* and goodness of fit *S* are based on *F*^2^, conventional *R*-factors *R* are based on *F*, with *F* set to zero for negative *F*^2^. The threshold expression of *F*^2^ > σ(*F*^2^) is used only for calculating *R*-factors(gt) *etc*. and is not relevant to the choice of reflections for refinement. *R*-factors based on *F*^2^ are statistically about twice as large as those based on *F*, and *R*- factors based on ALL data will be even larger.

### Fractional atomic coordinates and isotropic or equivalent isotropic displacement parameters (Å^2^)

**Table:**

	*x*	*y*	*z*	*U*_iso_*/*U*_eq_	
S1	−0.16661 (11)	0.67441 (7)	0.42200 (3)	0.0592 (2)	
O1	0.3364 (3)	0.2322 (2)	0.26744 (9)	0.0561 (6)	
O2	0.3978 (3)	0.3163 (2)	0.36803 (8)	0.0651 (7)	
O3	0.3730 (2)	0.55141 (18)	0.23747 (8)	0.0475 (5)	
O4	−0.2132 (3)	0.3346 (2)	0.21563 (10)	0.0656 (7)	
N1	0.0832 (3)	0.43417 (17)	0.24261 (8)	0.0355 (5)	
C1	0.2192 (3)	0.5168 (2)	0.21199 (11)	0.0361 (6)	
C2	0.1381 (4)	0.5487 (2)	0.14518 (10)	0.0390 (6)	
C3	0.2158 (5)	0.6255 (3)	0.09427 (12)	0.0543 (8)	
C4	0.1055 (5)	0.6347 (3)	0.03591 (13)	0.0638 (9)	
C5	−0.0735 (6)	0.5715 (3)	0.03025 (14)	0.0668 (9)	
C6	−0.1514 (5)	0.4944 (3)	0.08144 (13)	0.0594 (8)	
C7	−0.0415 (4)	0.4844 (2)	0.13909 (11)	0.0430 (6)	
C8	−0.0790 (3)	0.4072 (2)	0.20120 (11)	0.0422 (7)	
C9	0.1029 (3)	0.3829 (2)	0.31004 (10)	0.0358 (6)	
C10	0.0710 (3)	0.4911 (2)	0.36262 (11)	0.0423 (6)	
C11	−0.1370 (4)	0.5462 (3)	0.36027 (11)	0.0465 (7)	
C12	−0.4193 (6)	0.7176 (4)	0.40992 (18)	0.0927 (14)	
C13	0.2982 (3)	0.3089 (2)	0.31883 (11)	0.0412 (6)	
H1	0.435 (5)	0.196 (3)	0.2742 (16)	0.0673*	
H3	0.33622	0.66910	0.09873	0.0652*	
H4	0.15390	0.68438	0.00030	0.0764*	
H5	−0.14459	0.58059	−0.00904	0.0801*	
H6	−0.27220	0.45141	0.07712	0.0713*	
H9	0.002 (4)	0.316 (3)	0.3151 (12)	0.0430*	
H10A	0.09595	0.45360	0.40636	0.0507*	
H10B	0.16374	0.56373	0.35543	0.0507*	
H11A	−0.23038	0.47436	0.36834	0.0558*	
H11B	−0.16321	0.58325	0.31651	0.0558*	
H12A	−0.43704	0.75226	0.36576	0.1385*	
H12B	−0.45705	0.78452	0.44181	0.1385*	
H12C	−0.49950	0.63905	0.41568	0.1385*	

### Atomic displacement parameters (Å^2^)

**Table:**

	*U*^11^	*U*^22^	*U*^33^	*U*^12^	*U*^13^	*U*^23^
S1	0.0668 (4)	0.0560 (3)	0.0549 (4)	0.0046 (3)	0.0121 (3)	−0.0137 (3)
O1	0.0542 (10)	0.0646 (11)	0.0494 (10)	0.0238 (9)	−0.0114 (9)	−0.0182 (9)
O2	0.0685 (11)	0.0852 (14)	0.0417 (9)	0.0298 (11)	−0.0157 (8)	−0.0100 (9)
O3	0.0448 (8)	0.0545 (9)	0.0432 (9)	−0.0130 (8)	−0.0076 (7)	0.0070 (7)
O4	0.0510 (10)	0.0801 (13)	0.0656 (12)	−0.0252 (10)	−0.0100 (9)	0.0065 (11)
N1	0.0360 (8)	0.0381 (8)	0.0323 (8)	−0.0015 (8)	−0.0004 (7)	0.0012 (7)
C1	0.0399 (10)	0.0326 (9)	0.0359 (10)	0.0005 (8)	0.0000 (8)	−0.0018 (8)
C2	0.0508 (12)	0.0346 (9)	0.0315 (10)	0.0027 (9)	−0.0023 (9)	−0.0029 (8)
C3	0.0728 (17)	0.0508 (12)	0.0394 (12)	−0.0090 (12)	−0.0011 (12)	0.0059 (10)
C4	0.097 (2)	0.0591 (14)	0.0353 (12)	−0.0061 (16)	−0.0088 (14)	0.0057 (11)
C5	0.100 (2)	0.0601 (15)	0.0404 (13)	0.0027 (17)	−0.0261 (15)	0.0002 (12)
C6	0.0686 (16)	0.0573 (13)	0.0524 (14)	−0.0024 (14)	−0.0235 (14)	−0.0051 (12)
C7	0.0502 (12)	0.0398 (10)	0.0391 (11)	0.0021 (10)	−0.0066 (10)	−0.0046 (9)
C8	0.0398 (11)	0.0440 (11)	0.0427 (12)	−0.0016 (9)	−0.0058 (9)	−0.0035 (9)
C9	0.0382 (10)	0.0369 (10)	0.0324 (10)	0.0002 (9)	0.0022 (8)	0.0020 (8)
C10	0.0460 (12)	0.0467 (11)	0.0342 (10)	0.0049 (10)	0.0008 (9)	−0.0039 (9)
C11	0.0494 (12)	0.0517 (12)	0.0383 (11)	0.0096 (11)	0.0036 (10)	−0.0045 (10)
C12	0.089 (2)	0.111 (3)	0.078 (2)	0.053 (2)	0.0201 (19)	−0.001 (2)
C13	0.0466 (11)	0.0424 (10)	0.0347 (10)	0.0074 (10)	0.0002 (9)	0.0013 (9)

### Geometric parameters (Å, °)

**Table:**

S1—C11	1.792 (3)	C7—C8	1.488 (3)
S1—C12	1.786 (4)	C9—C13	1.528 (3)
O1—C13	1.311 (3)	C9—C10	1.525 (3)
O2—C13	1.200 (3)	C10—C11	1.516 (3)
O3—C1	1.213 (3)	C3—H3	0.9300
O4—C8	1.199 (3)	C4—H4	0.9300
O1—H1	0.77 (3)	C5—H5	0.9300
N1—C8	1.407 (3)	C6—H6	0.9300
N1—C9	1.454 (3)	C9—H9	0.96 (3)
N1—C1	1.382 (3)	C10—H10A	0.9700
C1—C2	1.486 (3)	C10—H10B	0.9700
C2—C7	1.383 (4)	C11—H11A	0.9700
C2—C3	1.382 (3)	C11—H11B	0.9700
C3—C4	1.395 (4)	C12—H12A	0.9600
C4—C5	1.374 (5)	C12—H12B	0.9600
C5—C6	1.389 (4)	C12—H12C	0.9600
C6—C7	1.382 (4)		
			
S1···C7^i^	3.610 (2)	C10···O3	3.301 (3)
S1···H5^ii^	3.1600	C11···C8	3.506 (3)
S1···H12B^iii^	3.1100	C13···O3	2.960 (3)
O1···N1	2.693 (3)	C1···H1^vi^	2.96 (3)
O1···C1	3.148 (3)	C1···H10B	2.9400
O1···O3^iv^	2.673 (3)	C2···H9^i^	2.94 (3)
O2···C4^v^	3.409 (3)	C3···H9^i^	3.02 (3)
O2···C3^iv^	3.328 (4)	C8···H11B	2.9600
O3···C13	2.960 (3)	H1···O3^iv^	1.96 (3)
O3···C10	3.301 (3)	H1···C1^iv^	2.96 (3)
O3···O4^i^	3.165 (3)	H3···O2^vi^	2.4200
O3···O1^vi^	2.673 (3)	H4···O2^viii^	2.6800
O4···O3^vii^	3.165 (3)	H5···S1^ix^	3.1600
O1···H12A^vii^	2.7700	H6···H12B^x^	2.5100
O1···H11B^vii^	2.5400	H9···O4	2.48 (3)
O2···H3^iv^	2.4200	H9···H11A	2.4700
O2···H10A	2.5800	H9···C2^vii^	2.94 (3)
O2···H4^v^	2.6800	H9···C3^vii^	3.02 (3)
O3···H10B	2.7700	H10A···O2	2.5800
O3···H1^vi^	1.96 (3)	H10B···O3	2.7700
O4···H9	2.48 (3)	H10B···C1	2.9400
N1···O1	2.693 (3)	H11A···H9	2.4700
N1···H11B	2.6900	H11B···N1	2.6900
C1···O1	3.148 (3)	H11B···C8	2.9600
C3···O2^vi^	3.328 (4)	H11B···O1^i^	2.5400
C4···O2^viii^	3.409 (3)	H12A···O1^i^	2.7700
C7···S1^vii^	3.610 (2)	H12B···S1^xi^	3.1100
C8···C11	3.506 (3)	H12B···H6^xii^	2.5100
			
C11—S1—C12	100.68 (15)	O1—C13—O2	125.0 (2)
C13—O1—H1	108 (2)	C2—C3—H3	122.00
C1—N1—C8	111.95 (17)	C4—C3—H3	122.00
C1—N1—C9	124.23 (18)	C3—C4—H4	119.00
C8—N1—C9	123.80 (18)	C5—C4—H4	120.00
O3—C1—N1	123.8 (2)	C4—C5—H5	119.00
O3—C1—C2	129.8 (2)	C6—C5—H5	119.00
N1—C1—C2	106.38 (18)	C5—C6—H6	121.00
C1—C2—C7	107.94 (18)	C7—C6—H6	121.00
C3—C2—C7	121.8 (2)	N1—C9—H9	106.0 (15)
C1—C2—C3	130.2 (2)	C10—C9—H9	108.4 (16)
C2—C3—C4	117.0 (3)	C13—C9—H9	105.8 (17)
C3—C4—C5	121.0 (3)	C9—C10—H10A	109.00
C4—C5—C6	122.0 (3)	C9—C10—H10B	109.00
C5—C6—C7	117.1 (3)	C11—C10—H10A	109.00
C2—C7—C8	108.42 (19)	C11—C10—H10B	109.00
C6—C7—C8	130.4 (2)	H10A—C10—H10B	108.00
C2—C7—C6	121.2 (2)	S1—C11—H11A	110.00
O4—C8—N1	124.6 (2)	S1—C11—H11B	110.00
N1—C8—C7	105.28 (18)	C10—C11—H11A	110.00
O4—C8—C7	130.1 (2)	C10—C11—H11B	110.00
N1—C9—C10	112.62 (16)	H11A—C11—H11B	108.00
C10—C9—C13	112.59 (17)	S1—C12—H12A	109.00
N1—C9—C13	110.92 (17)	S1—C12—H12B	109.00
C9—C10—C11	111.50 (18)	S1—C12—H12C	109.00
S1—C11—C10	109.93 (17)	H12A—C12—H12B	110.00
O1—C13—C9	111.22 (18)	H12A—C12—H12C	109.00
O2—C13—C9	123.7 (2)	H12B—C12—H12C	109.00
			
C12—S1—C11—C10	−178.69 (19)	C1—C2—C7—C8	−1.1 (2)
C8—N1—C1—O3	−178.4 (2)	C3—C2—C7—C6	0.0 (4)
C8—N1—C1—C2	1.0 (2)	C3—C2—C7—C8	178.1 (2)
C9—N1—C1—O3	2.7 (3)	C2—C3—C4—C5	1.0 (4)
C9—N1—C1—C2	−177.97 (18)	C3—C4—C5—C6	−1.0 (5)
C1—N1—C8—O4	177.4 (2)	C4—C5—C6—C7	0.5 (5)
C1—N1—C8—C7	−1.6 (2)	C5—C6—C7—C2	0.0 (4)
C9—N1—C8—O4	−3.6 (3)	C5—C6—C7—C8	−177.6 (3)
C9—N1—C8—C7	177.32 (18)	C2—C7—C8—O4	−177.3 (2)
C1—N1—C9—C10	72.2 (2)	C2—C7—C8—N1	1.7 (2)
C1—N1—C9—C13	−55.0 (2)	C6—C7—C8—O4	0.6 (4)
C8—N1—C9—C10	−106.6 (2)	C6—C7—C8—N1	179.6 (3)
C8—N1—C9—C13	126.1 (2)	N1—C9—C10—C11	63.5 (2)
O3—C1—C2—C3	0.3 (4)	C13—C9—C10—C11	−170.15 (18)
O3—C1—C2—C7	179.4 (2)	N1—C9—C13—O1	−42.6 (2)
N1—C1—C2—C3	−179.0 (2)	N1—C9—C13—O2	140.5 (2)
N1—C1—C2—C7	0.2 (2)	C10—C9—C13—O1	−169.85 (18)
C1—C2—C3—C4	178.6 (2)	C10—C9—C13—O2	13.3 (3)
C7—C2—C3—C4	−0.5 (4)	C9—C10—C11—S1	−179.19 (15)
C1—C2—C7—C6	−179.3 (2)		

Symmetry codes: (i) −*x*, *y*+1/2, −*z*+1/2; (ii) −*x*−1/2, −*y*+1, *z*+1/2; (iii) *x*+1/2, −*y*+3/2, −*z*+1; (iv) −*x*+1, *y*−1/2, −*z*+1/2; (v) −*x*+1/2, −*y*+1, *z*+1/2; (vi) −*x*+1, *y*+1/2, −*z*+1/2; (vii) −*x*, *y*−1/2, −*z*+1/2; (viii) −*x*+1/2, −*y*+1, *z*−1/2; (ix) −*x*−1/2, −*y*+1, *z*−1/2; (x) −*x*−1, *y*−1/2, −*z*+1/2; (xi) *x*−1/2, −*y*+3/2, −*z*+1; (xii) −*x*−1, *y*+1/2, −*z*+1/2.

### Hydrogen-bond geometry (Å, °)

**Table:**

*D*—H···*A*	*D*—H	H···*A*	*D*···*A*	*D*—H···*A*
O1—H1···O3^iv^	0.77 (3)	1.96 (3)	2.673 (3)	154 (3)
C3—H3···O2^vi^	0.9300	2.4200	3.328 (4)	165.00
C9—H9···O4	0.96 (3)	2.48 (3)	2.905 (3)	106.4 (18)
C11—H11B···O1^i^	0.9700	2.5400	3.443 (3)	156.00

Symmetry codes: (iv) −*x*+1, *y*−1/2, −*z*+1/2; (vi) −*x*+1, *y*+1/2, −*z*+1/2; (i) −*x*, *y*+1/2, −*z*+1/2.
